# Clinical Implications of Nanosciences in Dentistry and Periodontics: A Narrative Review

**DOI:** 10.7759/cureus.48593

**Published:** 2023-11-10

**Authors:** Ritiksha Agrawal, Pavan Bajaj, Unnati Shirbhate, Amit Reche, Abhishek Pahade, Arpit Barhate

**Affiliations:** 1 Dentistry, Sharad Pawar Dental College and Hospital, Datta Meghe Institute of Higher Education and Research (Deemed to be University), Wardha, IND; 2 Periodontics, Sharad Pawar Dental College and Hospital, Datta Meghe Institute of Higher Education and Research (Deemed to be University), Wardha, IND; 3 Public Health Dentistry, Sharad Pawar Dental College and Hospital, Datta Meghe Institute of Higher Education and Research (Deemed to be University), Wardha, IND

**Keywords:** nanoperiodontics, dental applications, nanomaterials, periodontics, dentistry, nanosciences

## Abstract

The nanosciences have recently emerged as a transformative force in dentistry and periodontics, offering fresh strategies to further the development of dental care. This paper provides a concise summary of the effect of nanoparticles, their categorisation, several methods of action, and various dental uses. This review discusses the properties of nanoparticles that lend to their use in dentistry and traces the history of the growth and advancement of nanotechnology in this area, nanomaterials' role in improving dental restorations' durability, aesthetics, and overall dental health by drawing on particular examples from restorative dentistry, prosthodontics, cosmetic dentistry, and general dentistry. In addition, the advancement of nanosciences has made periodontal regeneration easier, which has resulted in more accurate forecasting of its effects. Issues relating to safety, finances, and regulations imposed by the government have been fixed. It is encouraged that research be conducted into the full potential of nanosciences in dentistry and periodontics as a method of realising the field's bright future. Applications of nanotechnology in dentistry and periodontics can be broadly discussed under the prevention, detection, and treatment modalities.

## Introduction and background

The application of nanosciences to dental and periodontal care offers a paradigm shift that promises novel approaches and fruitful results. New opportunities for precision and efficiency in dental applications have been made possible by nanotechnology, which operates at the nanoscale. This article highlights nanosciences' importance to oral healthcare's future by providing insight into their background, various applications, and potential prospects. It can't be early as nanotechnology has been in use for the past many years in dentistry [1]. The use of nanomaterials and procedures for oral health heralded a new age. Researchers pioneered nanodentistry in the early years, laying the groundwork for later [1,2]. Key historical developments and turning points have played a significant role in moulding the present [2]. The implications of using nanotechnology in dentistry are far-reaching. Indicative of a shift towards individualised dental care, recent studies highlight the possibility of controlled local medication delivery systems [3]. More so, bioactive glass nanoparticles have been studied, and they show great promise for periodontal regeneration and dental biomaterial applications. Dental health and nanomaterials have worked together to improve tooth strength and appearance and general dental health [4].

Using the abundance of research from the numerous sources cited in this study, this review aims to provide a comprehensive examination of the uses of nanosciences in dentistry and periodontics. This review aims to shed light on the current state of nanotechnology by reviewing its historical context, identifying nanomaterials, elaborating on their mode of action, discussing their characteristics, and exploring their applications in dental and periodontal operations. Dental nanotechnology's difficulties, potential toxicological worries, and regulatory factors will be explored as well. Finally, we'll look ahead to the promising future of nanosciences in dentistry and periodontics and discuss some of the fascinating potential.

## Review

History

Several pivotal moments, such as pulp repair, periodontal ligament regeneration, bio-nano surface technology, and implant osseointegration, have occurred in nanotechnology's application to dentistry. The groundbreaking work of established nanodentistry is a new and exciting area of study. Nanotechnology set the path for additional research into the potential benefits of using nanomaterials and techniques in dental treatment [5]. According to early research efforts in developing the concept of nanodentistry, it paved the way for additional in-depth study in this field, which led to significant new advances and laid the framework for future advancements by examining nanoscale materials and their possible applications [2]. As the field of nanotechnology developed, researchers began to investigate its similarities to dental science. An excellent example of this synergy aimed to usher in a new era of personalised dental care by studying controlled local drug delivery systems. Dental biomaterials and periodontal regeneration treatments, including bioactive glass nanoparticles, have been found to have potential in tissue regeneration [6-8].

These seminal accomplishments paved the way for the application of nanotechnology in dentistry. Since its infancy, the field has matured to include numerous applications with promising potential to advance oral healthcare by utilising nanomaterials and other novel techniques. Since its first investigations, nanodentistry has gone a long way, and its most recent successes have opened up promising new paths for the future of oral healthcare.

Classification

Multiple nanomaterials with unique features and applications are used in nanodentistry. In dentistry, nanomaterials typically fall into one of the three categories: nanoparticles, nanocomposites, and nanostructured materials. To fully appreciate their roles and opportunities in the industry, they must have a clear grip on these classifications. Nanoparticles are a crucial part of the application of nanotechnology in dentistry. They range in size from 1 to 100 nanometers and are incredibly tiny, isolated entities. As a result of their unique properties at the nanoscale, nanoparticles find widespread usage in fields as diverse as dentistry as drug delivery systems, diagnostic agents, and tissue engineering. Due to their adaptability and versatility, nanoparticles play a critical role in enhancing the precision and efficiency of dental treatments [9]. Nanocomposites are a class of novel materials combining traditional dental materials like resins and ceramics with nanoparticles [10,11]. The enhanced durability, resistance to germs, and appealing look of nanoparticles are exploited in these nanocomposites. Cosmetic and restorative dentists employ them to give patients long-lasting, aesthetically pleasing outcomes.

Research indicates that nanostructured materials have a distinct nanoscale structure [12]. Mechanical properties aside, these materials also offer exceptional overall performance. Since nanostructured materials provide enhanced osseointegration and durability, they are increasingly being used in the fabrication of dental implants. Understanding the differences between these categories is critical for dentists in choosing the most effective nanomaterial for a particular application. Nanocomposites enhance restorative quality and durability, nanoparticles permit targeted drug delivery, and nanostructured materials boost dental implant function. Dentists can better serve their patients using nanotechnology if they know the distinctions between these types.

Mechanism of action

Fundamental principles that control the behaviour of nanoparticles underpin the application of nanotechnology in dentistry and periodontics. Because of these guidelines, dental tools and techniques are now more effective than ever. The mechanism of action of nanoparticles in dentistry is based on their distinctive features. Changing physical and chemical characteristics at the nanoscale boosts reactivity and surface area in materials [9]. This quality allows oral drug delivery devices to have exact control over drug release rates. An effective strategy in periodontics is using nanoparticles because of their regulated release of therapeutic drugs [3]. In addition, nanoparticles have played a crucial role in improving the functionality of dental materials. Nanoparticles added to composites enhance their mechanical properties, leading to restorations with increased longevity and resilience [7]. As described in detail, the nanoscale architecture of nanostructured materials aids the osseointegration and stability of dental implants [12,13].

The incorporation of nanotechnology has revolutionised the detection and treatment of periodontal diseases. Nanoscale diagnostic agents can reliably identify biofilm and bacterial infections in periodontal pockets. Broad-spectrum antibiotics can be decreased, and therapeutic efficacy can be improved because of this precise diagnosis [10]. Nanomaterials' mode of action in dentistry and periodontics, driven by their distinctive qualities and precision, has ushered in a new era of individualised and potent dental care. Nanotechnology has the potential to radically alter how dental practitioners do diagnostics, treat patients, and provide aftercare [14,15].

Applications in dentistry

Nanotechnology's versatility has allowed it to spread throughout the field of dentistry, opening up exciting new avenues for enhancing both patient care and professional practice. The advent of nanosciences has opened up a wealth of possibilities for dentists in general practice. It demonstrated that nanoparticles have significantly impacted restorative dentistry [16-19]. Nanocomposites, used in dental restorations and consisting of nanoparticles combined with traditional dental materials, have become increasingly popular in recent years. It has been proven that mechanical properties like strength and durability increase [20]. Improvements in nanotechnology have allowed dental materials to mimic the appearance of natural teeth more closely. Nanotechnology has revolutionised the field of prosthodontics, which deals with restoring teeth that have been lost. Nanostructured materials have enhanced the performance and osseointegration of dental implants, a cornerstone of prosthodontic care [6]. The nanoscale organisation of these materials aids in their integration with bone, increasing dental implants' durability and stability. This has far-reaching consequences for patients needing prosthetic teeth, as it provides a more stable and durable alternative.

Nanotechnology has ushered in a new era of smile enhancement in cosmetic dentistry. Nanomaterials play an essential role in dental whitening therapies. Nanoparticles are crucial for effectively delivering tooth-whitening chemicals, leading to less sensitivity and better aesthetic results. Patients can expect their veneers and crowns to look and feel like their natural teeth due to nanomaterials' usage in cosmetic dentistry [17]. As demonstrated, nanomaterials also play a role in creating new diagnostic tools for general dentistry. It is now possible to detect biofilm and bacterial infections in periodontal pockets using nanoscale diagnostic agents. This accuracy level makes targeted treatment possible by decreasing broad-spectrum antibiotics and increasing treatment efficacy [18-21]. The various ways in which nanosciences are used in general dentistry demonstrate the revolutionary potential of nanotechnology to improve dental treatment for patients, clinical outcomes, and the profession as a whole. Nanomaterials provide individualised treatments, enhanced performance, and aesthetic upgrades in all areas of dentistry, leading to better dental health and greater patient satisfaction.

Properties

Because of their unique features, nanomaterials are ideally suited for use in dentistry, which will lead to significant advances in this field. Nanotechnology's adaptability and efficacy in dentistry came from its size, surface area, and reactivity. Nanomaterials stand out due to their minuscule size. At the nanoscale, the size of materials can be anywhere from 1 to 100 nanometers [15,22-25]. Due to their tiny size, nanoparticles have more precision and mobility in the mouth. For instance, nanoparticles utilised in dental drug delivery systems can accurately target specific areas within the mouth cavity. This size-specific accuracy is crucial to individualised dental care [14].

Nanomaterials are distinguished by their large surface area relative to their small volume. Bioactive glass nanoparticles in periodontal regeneration have been shown to provide significant therapeutic effects because of their high surface area, allowing efficient interactions with surrounding tissues and fluids [26]. Surface area increases bioactivity and facilitates interactions with the host tissue, speeding up the regeneration process. At the nanoscale, another essential feature, reactivity, is amplified. Nanomaterials changed chemical and physical characteristics, leading to increased reactivity. The ability of nanoparticles to release therapeutic drugs in a controlled and sustained manner is exploited in dental medication delivery systems to guarantee efficient therapy with few adverse effects. In treating oral infections and inflammation, a high degree of responsiveness is invaluable [27-29].

Dental techniques and materials that use nanotechnology make use of these one-of-a-kind qualities. All of these qualities, especially the nanoscale size that permits precise interventions and the high surface area that boosts bioactivity and reactivity, contribute to the accuracy and efficacy of dental treatments. To fully realise the promise of nanotechnology in dental applications, it is essential to understand these features.

Development of Nanotechnology in the Field of Dentistry

Pioneering research and development efforts have led to extraordinary growth and evolution in applying nanotechnology in dentistry. Nanotechnology has brought revolutionary changes in dentistry and oral healthcare [30]. They paved the way for future study in the field of oral healthcare by investigating the use of nanomaterials and methods. The further exploration of nanodentistry paved the way for more profound studies. The potential of nanoparticles in dentistry was defined by this preliminary investigation [31]. The advancement of dental nanosciences can be directly attributed to research and development efforts. Controlled local drug delivery systems have been developed, an essential advance in individualised dental care [27,28]. The evolution of nanotechnology in dentistry has been marked by a relentless pursuit of better patient care, more effective treatments, and uncharted territory. Improved medicine delivery systems and longer-lasting dental materials are just two examples of the many advances made possible by research and development activities. Research and development have significantly impacted the contemporary landscape of dental nanosciences, as evidenced by the action of nanotechnology in the area.

Nanotechnology has changed the face of many dental treatments by providing cutting-edge options to enhance teeth hygiene, aesthetics, and longevity. Specific nanomaterials have been crucial to developing these applications, improving the accuracy and efficiency of dental care. The use of nanomaterials in this field of dentistry is on the rise. For instance, nanocomposites, which combine nanoparticles with conventional dental materials, result in fixes that are not only more durable but also aesthetically pleasing. Mechanical characteristics and aesthetics are enhanced through nanocomposites to create a restoration that looks and functions like the patient's natural teeth [26]. Dental implants are a mainstay of prosthodontic care, and nanotechnology has significantly impacted them. The well-ordered nanoscale structure of nanostructured materials reportedly allows for better osseointegration [25]. Improvements in implant stability and integration with surrounding bone tissue have ushered in a new era of reliability and longevity for those with prosthetic teeth.

The advent of nanotechnology in cosmetic dentistry has allowed for more aesthetically pleasing procedures. Targeted medication delivery by nanoparticles has been used in teeth-whitening treatments, as reported by Qasim and Rehman [24]. The method of tooth whitening has been made more effective and less sensitive, guaranteeing patients the most excellent possible cosmetic results. Biofilm and bacterial infections in periodontal pockets can be accurately detected using nanoscale diagnostic agents. Improved therapeutic outcomes have been observed alongside a reduced requirement for systemic antibiotics, thanks to targeted treatment made feasible by the precise diagnostics provided by nanoscale agents. The accuracy of this order is essential for effectively managing oral infections and inflammation [23].

Nanotechnology in dentistry has dramatically improved the speed and precision of dental care. Dental health, durability, and aesthetics can all benefit from nanomaterials, which can be applied across many different areas of dentistry. These applications demonstrate the potential of nanotechnology to improve dental treatment for patients.

Applications of nanoperiodontics

The application of nanotechnology in periodontics is referred to as nanoperiodontics. Nanotechnology has revolutionised the treatment of periodontal disorders by allowing for more precise diagnosis and more effective restoration. Nanotechnology is now routinely used in the diagnosis of periodontal disease. Using nanoscale diagnostic agents, biofilm and bacterial infections in periodontal pockets have been successfully identified [22]. Because of their small size and high reactivity, pathogenic microorganisms are easy targets for these medicines. A correct diagnosis allows dental specialists to tailor treatment strategies to the bacteria implicated, reducing the need for broad-spectrum antibiotics and maintaining healthy oral flora.

The science of periodontal regeneration has made significant advancements because of nanotechnology. Bioactive glass nanoparticles have been shown to aid in periodontal regeneration [21]. The nanoscale order of these particles allows for more efficient communication with the host. This, in turn, helps restore periodontal tissues and speed up the regeneration process. Nanomaterials used in periodontal regeneration have improved outcome predictability and decreased patient morbidity. Nanoperiodontics is a subspecialty of periodontics that exemplifies how nanotechnology might enhance dental care. It aids in accurate diagnosis, which permits targeted treatments that zero in on specific bacteria. It also helps the regeneration process by speeding up the restoration of periodontal tissue. Fuelled by nanoscience, these technologies may drastically alter how periodontal diseases are treated, leading to better dental health and more favourable patient outcomes [17]. 

**Figure 1:**
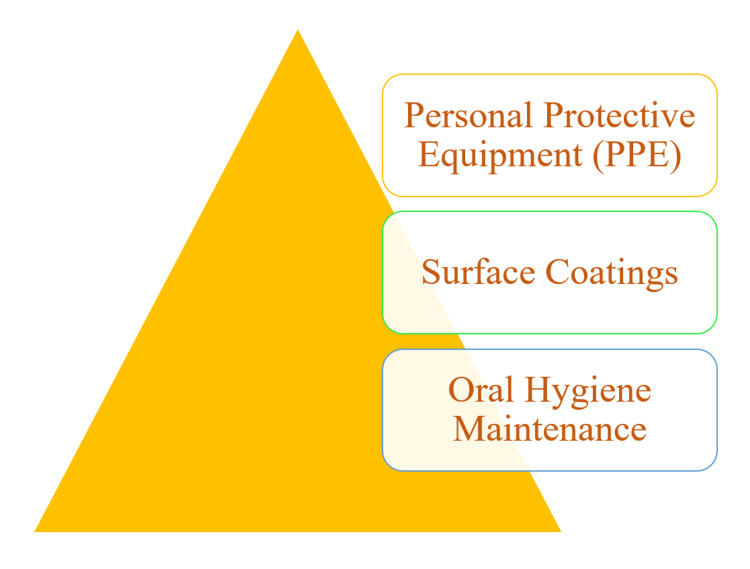
The prevention category comprises the following nanotechnology techniques Image Credit: Author

The broad categories of prevention, detection, and treatment can be used to address nanotechnology applications in periodontics. The prevention category comprises oral hygiene maintenance, surface coatings, and personal protective equipment (PPE) listed below in Figure 1.

**Figure 2:**
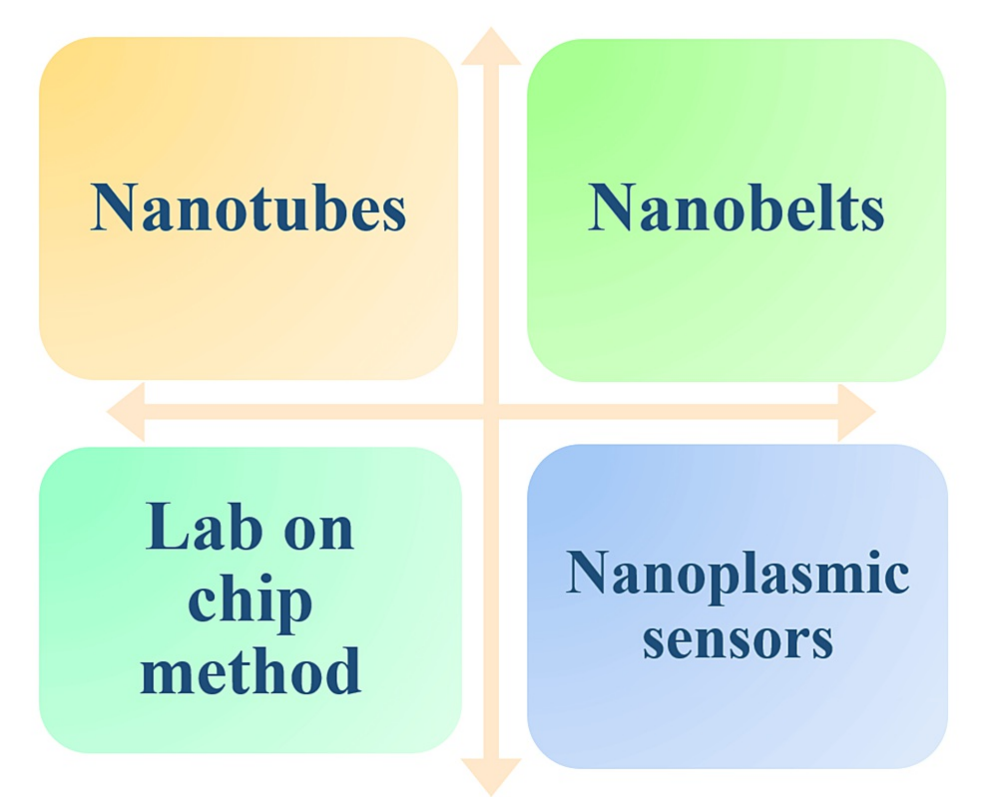
Diagnostic kits for the detection category use the following nanotechnology therapeutic techniques Image Credit: Author

Antimicrobial compounds produced from nanoparticles have superior effects because of their vast surface area, such as silica, silver, copper, and zirconia. Diagnostic kits based on nanotechnology are more effective than their original counterparts; they are also precise, sensitive, and portable [31-35]. Compared to their original image, diagnostic kits based on nanotechnology are more effective in the detection category; they are also more easily portable, sensitive, and specific, as listed in Figure 2.

The following uses of nanotechnology therapeutic techniques are displayed in Table 1. Dentifrobots can remove organic residues from the supragingival and subgingival surfaces by moving across the said surfaces, breaking down restricted organic materials into harmless fumes, and continuously removing calculus from the surface of the occlusal teeth [34]. Once ingested, these nanorobots securely self-deactivate and can travel at 1-10 microsecond (μ/s). Core-shell spheres, hollow spheres, nanotubes, and nanocomposites are the commonly employed nanomaterials for controlled drug delivery. Pharmaceuticals can be added to nanospheres made of a biodegradable polymer. Nanotechnology has been used in drug delivery because of its enhanced biocompatibility, targeted release, lowered antimicrobial resistance, extended half-life, and reduced toxicity [34,35].

**Table 1:** The treatment category uses the following nanotechnology therapeutic techniques References: [34,35]

Dentinal hypersensitivity	Subgingival irrigation	Local drug delivery
Dental longevity and maquillage	Laser and nanoparticles	Nanoscale particles in dental implants
Host immunomodulation therapy	Tissue engineering	Peri-implantitis
Bone grafts	Biofilm management	Self-assembling implants
Nanomembranes	Nanoantibiotics	Nanotweezers
Nanoneedles	Wound healing	Local anaesthesia

The process of nanoencapsulation involves encasing a bioactive chemical (BAC) in a matrix or inert medium, whether it be liquid, solid, or gaseous, to preserve the coated material. Southwest Research Institute (SWRI) recently created the nanoencapsulation process, a method for administering vaccinations and medicines. Liposomes, micelles, dendrimers, polymers, nanorattles, nanowires, and niosomes are a few examples of different drug delivery systems. Chitosan nanoparticles are an effective delivery vehicle in triclosan-based hydrogel nanocomposite delivery systems. Polydopamine (PDA) was coated onto nanofibrous membranes made of polycaprolactone (ε-caprolactone), a biocompatible polymer. The coating attracted calcium and phosphate ions, causing early bone mineralisation [32].

It is believed that nanotechnology-based grafts yield better results and can be utilised for sinus augmentation, socket preservation, and treating intrabony defects. These days, nanoscale particles are being used to develop alloplastic bone transplants. Because of its biocompatibility, nano-hydroxyapatite (nano-HA) is a material with several applications that elicits better cellular responses than "plain" chitosan scaffolds, the host-modifying drugs' immunomodulatory when administered through systems based on nanotechnology [17,31].

Toxicology in Nanodentistry

Nanomaterial use in dentistry has led to exciting new developments, yet there is still reason for concern about their safety and potential toxicity. Thorough research is necessary to allay fears about the use of nanoparticles in dentistry. Toxicology discusses the significance of comprehensive safety studies before introducing any nanodental product into clinical practice. The possibility of cytotoxicity and inflammation in oral tissues must be considered in these analyses [31]. The safe application of nanotechnology in dentistry is dependent on regulatory considerations. Regulatory agencies should create severe rules for approving and monitoring nanodental products, as pointed out by Garg et al. [32]. Regulators monitor nanomaterials to ensure they are devoid of harmful substances and adhere to strict safety guidelines.

Furthermore, the possible hazards of dental nanotechnology must be recognised and published to highlight the need to identify and address potential dangers linked with dental nanoparticles. Nanomaterial buildup in oral tissues and the formation of hazardous byproducts during degradation are two examples of possible unexpected consequences. Toxicology in nanodentistry is a developing field that needs careful study and oversight from regulators. Using nanotechnology in dentistry could significantly improve patient care, but there must be no compromise on safety. Harnessing the full potential of dental nanotechnology while protecting patient safety requires comprehensive toxicological assessments, tight regulatory guidelines, and risk appraisal [30].

Future scope

The field of nanosciences in dentistry is heading towards an exciting future full of new developments and directions. These breakthroughs open up promising new avenues for study and development.

Individualised Care

Nanotechnology's bright future in dentistry could one day allow for fully individualised treatment plans. A recent study hints at using bioactive glass nanoparticles to tailor therapy to an individual's oral health profile. Precision diagnostics and individualised treatments will likely become the norm [33]. Nanorobots are a promising new direction in nanodentistry. These microscopic tools can manoeuvre around the mouth and directly address specific tooth problems. Minimally invasive surgeries and better therapeutic results are possible with the help of nanorobots.

In the field of drug delivery, developments like those described by nanodental drug delivery devices are likely to continue. Drug delivery systems that react to local oral conditions may be developed to reduce unnecessary medication exposure, improve patient compliance, and decrease adverse effects [34]. Powered by nanotechnology, periodontal regeneration has great promise for future research into regenerative therapies. Using nanoparticles in regenerative therapies shows promise for the predictable and successful revitalisation of injured periodontal tissues [35]. In conclusion, nanosciences present a wealth of opportunity for the dental industry in the future. A few examples of what's to come in medicine are personalised care, nanorobots, cutting-edge drug delivery systems, and restorative therapies. Prospects for further enhancing patient care and oral health through nanotechnology are intriguing for researchers and dental practitioners.

Discussion

Nanodentistry has the potential to revolutionise dentistry, and this review highlights its many promising uses. Precision in a variety of dental operations is made possible by nanomaterials. They improve the restorative materials' mechanical qualities, which helps dental restorations last longer. Nanostructured materials improve osseointegration in prosthodontics, prolonging dental implant life. As a result of nanoparticle-driven precise drug delivery, tooth-whitening procedures are more comfortable and produce better cosmetic results with less sensitivity. Dental nanosciences, due to their interdisciplinary character, hold the promise of individualised care. Nanotechnology is a crucial component in the emerging field of highly personalised dental care because of its capacity to customise treatment plans based on patient's unique oral health profiles. One particularly promising application of nanoparticles is in periodontal regeneration. Periodontal health can be restored partly due to nanostructured materials and nanoscale diagnostic agents, increasing tissue recovery predictability [33-35].

Challenges and Limitations

The use of nanoparticles in dentistry raises questions about their safety. Potential side effects, such as cytotoxicity and inflammation, must be studied in depth. Regulatory considerations and risk evaluation are essential to guarantee the security of nanodental products. Concerns about treatment cost and availability have been voiced in response to the growing use of nanotechnology in dentistry. To ensure that all people, regardless of income, have access to the most cutting-edge dental care, it is essential to balance innovation, affordability, and accessibility as dental materials progress. Nanotechnology in dentistry is a prime example of the multidisciplinary character of modern medicine. It connects the fields of dentistry and materials science to those of nanotechnology and robotics. The use of nanorobots is an excellent example of the multidisciplinary effort required to advance dental treatment [21,22].

Furthermore, dental nanosciences highlight the significance of collaboration between academics, doctors, and regulatory authorities from different fields. Nanodentistry can only progress and adapt to the ever-changing oral healthcare landscape with the help of such partnerships. Nanodentistry, as this paper explains, is more than merely the application of nanoparticles to dental operations; rather, it represents a fundamental change in our approach to oral healthcare. It could improve accuracy, individualisation, and efficacy in medical care. However, the potential of dental nanosciences can only be realised by overcoming obstacles linked to safety and cost [33].

## Conclusions

The review has shown how dentistry and periodontics can benefit significantly from nanosciences. Precision and individualisation made possible by nanomaterials improve the longevity, cosmetics, and overall dental health of patients undergoing dental procedures. Nanotechnology plays a crucial role in periodontal regeneration with its ability to restore periodontal tissues predictably and effectively. Nanodentistry shows promise, but questions regarding its safety and cost still need answering, highlighting the importance of regulatory supervision and risk assessment. The possibility for novel, patient-centred treatments makes nanosciences in dentistry and periodontics so exciting for the future. Because of the dental nanosciences' interdisciplinary nature, cooperation among researchers, doctors, and authorities is essential. Constant study in this area is crucial. Ultimately, it will enhance patient care and dental health by refining existing applications and opening new avenues. Nanotechnology in dentistry promises to revolutionise dental care and oral health.
